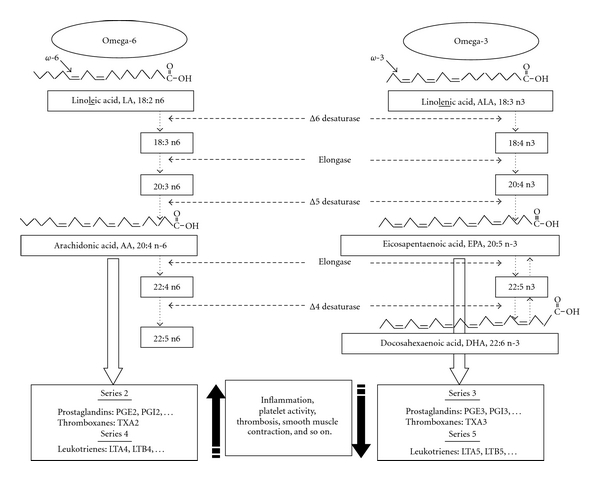# Retina and Omega-3

**DOI:** 10.1155/2011/748361

**Published:** 2011-10-31

**Authors:** Giuseppe Querques, Raimondo Forte, Eric H. Souied

**Affiliations:** Créteil University Eye Clinic, 40 Avenue de Verdun, 94000 Créteil, France

## Abstract

Over the last decade, several epidemiological studies based on food frequency questionnaires suggest that omega-3 polyunsaturated fatty acids could have a protective role in reducing the onset and progression of retinal diseases. The retina has a high concentration of omega-3, particularly DHA, which optimizes fluidity of photoreceptor membranes, retinal integrity, and visual function. Furthermore, many studies demonstrated that DHA has a protective, for example antiapoptotic, role in the retina. From a nutritional point of view, it is known that western populations, particularly aged individuals, have a higher than optimal omega-6/omega-3 ratio and should enrich their diet with more fish consumption or have DHA supplementation. This paper underscores the potential beneficial effect of omega-3 fatty acids on retinal diseases.

## 1. Introduction

Fatty acids are commonly classified as saturated, monounsaturated, and polyunsaturated fats. Saturated fats are a chain of carbon atoms joined by single links (butter, lard, and fat in meat). Monounsaturated fatty acids (MUFAs) are made up of a chain of carbon atoms containing one double bond (olive oil). Polyunsaturated fatty acids (PUFAs) contain at least two double bonds (Figure 1) and are classified by the position of the first double bound counting from the methyl terminal. When the first double bond is 6 carbon atoms from the methyl terminal, PUFAs are called omega-6, *ω*6, or n-6, and when the first double bound is 3 carbon atoms from the methyl terminal, PUFAs are called omega-3, *ω*3, or n-3 (Figure 1). Two PUFAs, linoleic acid (LA, C18:2n-6), an omega-6 fatty acid, and *α*-linolenic acid (ALA, C18:3n-3), an omega-3 fatty acid, are termed essential because they are required for optimal health, but cannot be synthesized by the body. Therefore, these essential fatty acids have to be supplied by the diet. These omega-6 and omega-3 essential fatty acids can be used by the body either as an energy source or be transformed with additional bounds, using elongase and desaturase enzymes into longer-chain PUFAs (LC PUFAs). However, the endogenous synthesis of LC PUFAs from the essential precursors LA and ALA is limited, and the diet needs to supply appropriate amounts of LC PUFAs. Among omega-6, LA is found mainly in vegetable products (soybean, sunflower, safflower, corn oils) and the major omega-6 LC-PUFA, the arachidonic acid (AA, C20:4n-6) is present in animal products (meat and egg yolk). Among omega-3, ALA is found mainly in vegetable products (rapeseed, canola, and nut oils). Key omega-3 LC PUFAs include eicosapentaenoic acid (EPA, C20:5n-3) and docosahexanoic acid (DHA, C22:6n-3) (Figure 1), both found primarily in oily cold-water fish such as tuna, salmon, sardines, and mackerel. Aside from fresh seaweed, a staple of many cultures, plant foods rarely contain EPA or DHA. 

## 2. Dietary Intake and Recommendations

Both omega-6 and omega-3 fatty acids are essential, but the body requires them in a ratio that is not normally achieved by the typical diet of today's industrialized nations. Experts think that man evolved on a diet which would have had roughly 1-2 times more omega-6 than omega-3, though there is a school of thought which argues for a 1 : 1 ratio [1]. Alaskan Eskimos, whose diet is rich in fish and marine mammals, have a plasma omega-6/omega-3 PUFA ratio of 3.5 : 1 [2]. Interestingly, lower rates of cardiovascular disease and atherosclerosis have been well documented in this native population [3, 4]. Currently, average intakes in Western Countries are in a ratio of around 8 : 1 to 20 : 1 in favour of the omega-6. The dietary ratio of omega-6/omega-3 consumed in the United States is 10.6 : 1 [5]. For Western societies, there are recommendations to decrease the intake of omega-6 PUFAs and increase the intake of omega-3 PUFAs. The World Health Organization (WHO) recommends the consumption of 0.3–0.5 g/day [6], while the International Society for the Study of Fatty Acids and Lipids (ISSFAL) advocates 500 mg/day [7], and the North Atlantic Treaty Organization (NATO) recommends 800 mg/day [8]. The Food and Drug Administration (FDA) and American heart association attribute the cardiovascular benefits of PUFAs to EPA and DHA and recommend a regular intake of *ω*3 LC PUFA intake of 0.9–1.0 g/day. A recent publication by the European Food Safety Authority (EFSA) postulates that the amount of EPA+DHA required to lower triglyceride levels is 2–4 g/day, and 3 g/day to lower blood pressure [9]. Dietary recommendations for *ω*3 LC PUFA intake for healthy adults have been set at a minimum of 650 mg/d by the International Society for the Study of Fatty Acids and Lipids [10]. This organisation recommends intake of DHA for adults to be at least 219 mg per day and 300 mg per day for pregnant and lactating women. The British Nutrition Foundation recommends 8 g EPA plus DHA per week for women (i.e., 1145 mg per day) or 10 g EPA plus DHA per week for men (i.e., 1430 mg per day) [11]. DACH association (Association of German, Austrian and Swiss Societies for Nutrition) recommends an ideal linoleic acid (omega-6 fatty acid)/alpha-linolenic acid (omega-3 fatty acid) ratio of 5 : 1 in the dietary intake [12].

The maximum tolerated dose of EPA plus DHA has been determined for humans at 300 mg/kg of body weight per day, which for an average-sized man (150 lbs) is approximately 22 g/day [13].

## 3. Omega-3 in the Retina

### 3.1. Structural Role of Omega-3 in the Retina

Essential fatty acids are structural components of all tissues and are indispensable for cell membrane synthesis. The structural lipids of the disk membranes consist primarily of phospholipids (80–90% of the total lipid) with low levels of cholesterol (8–10%), a composition that makes them unusually fluid. In vertebrates, although DHA represents a small percentage of the fatty acids in most tissues (1–5%), it accounts for approximately 50–60% of the total fatty acid content within rod outer segments of photoreceptors (ROSs) [14–16]. It is demonstrated in piglets that supplementation of dietary DHA significantly increase DHA in brain and retina [17, 18]. To compensate for the continuous oxidative damage, the photoreceptors have the capacity of continuous renewal. While new outer segment disks are being generated at the base of the ROS, old disks at the tip are being phagocytosed by the cells of the RPE, which also have a very active metabolism, but unlike the photoreceptors do not have any potential to regenerate so that damaged cells cannot be replaced [19]. In the retina, DHA is mainly but not exclusively located in photoreceptor cells, where it becomes esterified into phospholipids. It has been thought to play an important role for the synthesis of disk membranes, in providing an adequate environment for conformational rhodopsin changes and in modifying the activity of retinal enzymes. Approximately, 60 molecules of phospholipids surround each molecule of visual pigment. Comparisons between DHA and saturated lipids in ROS membranes showed that PUFA favor chain extension while maintaining bilayer flexibility and have a tendency to adopt a hairpin-like structure, which increased the interfacial area per lipid [20]. Another role is the renewal of outer segments, which proceeds by the shedding of discs and the resynthesis of new membranes [21]. A deficiency of DHA in the membranes of photoreceptors disturbs membrane fluidity and function and could alter the process of outer segment renewal [22]. DHA is also suggested to play a role in early photoreceptor differentiation [23].

### 3.2. Functional Role of DHA in the Retina

The composition of the membrane lipid bilayer has a direct influence on the energy of the conformational states of rhodopsin in visual excitation [14, 24]. Bush et al. demonstrated that DHA plays an important role in the regeneration of rhodospin. An 80% reduction in retinal DHA level in omega-3 deficient rats is associated with a significantly slower rate of rhodopsin regeneration after 100% bleach. In fact, interactions between DHA and rhodopsin occur as early as their cotransport in the post-Golgi vesicules to ROS [14]. The dependency on docosahexaenoic acid-containing phospholipid bilayers has recently been described for the kinetic conversion of metarhodopsin I to metarhodopsin II [25], the formation of activated rhodopsin-transducin complex [26], and the activation of phosphodiesterase [27]. Furthermore, DHA acyl chains promote the coupling of metarhodopsin II to the retinal G protein [28]. DHA has also significant effects on photoreceptor membranes and neurotransmitters involved in the signal transduction process, rhodopsin activation, rod and cone development, neural dendritic connectivity, and functional maturation of the central nervous system [29]. A concentration gradient of DHA normally exists in the subretinal space between the rod outer segments (higher concentration) and the retinal pigment epithelium (lower concentration), and the release of 11-*cis* retinal from interphotoreceptor retinoid-binding protein (IRBP) is facilitated when IRBP is exposed to sufficient DHA in the subretinal space [30, 31]. 

The role of 22:6n-3 in the retina has not been exactly defined, although dietary deprivation of this essential fatty acid or its precursors leads to changes in retinal function in rats, cats, guinea pigs, and monkeys [32–40]. Benolken et al. demonstrated that rats fed a fat-free diet for two generations had abnormally low DHA levels in photoreceptor membranes and low ERG amplitudes [39]. The amplitude of this light-evoked retinal response increased in proportion to the amount of omega-3 PUFAs added to the diet. In third-generation omega-3 PUFA-deficient rats, DHA levels in retina were reduced by 55% [40]. The omega-3-deficient rats exhibited significantly reduced retinal sensitivity and increased b-wave implicit times compared with those fed the n-3-adequate diet. 

Carrié et al. investigated the effects of DHA deprivation and supplementation of DHA on behavior, ERG and PUFA composition in the brain, and retina of old mice [41]. In deficient mice, supplementation with phospholipids rich in DHA restored retinal level of DHA and amplified the b-wave amplitude on ERG. Dietary provision of DHA during aging improved the visual abilities both in control and deficient mice. This study suggests a potential reversibility of retinal dysfunction linked with omega-3 deficiency, even in aged animals. 

The guinea pig provides a model in which dietary omega-3 fatty acid deficiency could produce significant reductions in the retinal omega-3 profile in a single generation. In guinea pigs, DHA deprivation induces a significant decrease in retinal DHA and decrease in b- and a-waves ERG amplitudes [36]. However, 10-week repletion allowed a complete functional recovery of both receptoral and postreceptoral components of the ERG responses [36]. It is notable that over a certain amount of DHA, essential for normal retinal function, an increase in the retinal DHA level past an optimal amount (around 19%) and seems to provide diminishing returns [35].

Neuringer et al. fed two cohorts of rhesus monkeys diets that contained either 0.3% ALA (low ALA) or 8% ALA (high ALA) as the sole omega-3 fatty acid [42, 43]. A second cohort also included a third dietary group supplemented with LC-PUFAs (DHA 0.6%, EPA 0.2%, and AA 0.2%). ERGs were recorded from the monkeys as infants (3-4 months), juveniles (1-2 years), and the second cohort as young adults (4–6 years). In the first cohort, low-ALA monkeys had reduced rod and cone ERG a-wave amplitudes as infants compared with the high-ALA monkeys. These reduced amplitudes were not observed in the second cohort when tested as juveniles or adults. The most marked and consistent alteration in retinal function in monkeys fed with the low ALA diet was a delay in the time required for the rod photoreceptors to recover after a light flash. The reduction in ERG amplitude at short intervals between flashes suggested a delay in recovery of the rod photoreceptors, a conclusion recently confirmed in adult rhesus monkeys by measuring rod recovery [44, 45]. 

To summarize, studies in mice, rats, guinea pigs, cats, and rhesus monkeys highlight important species differences with respect to the effect of reducing retinal DHA levels on retinal function. It is unclear why these species should exhibit such contrasting ERG alterations in response to an omega-3 deficient diet. However, the omega-3 deficient diets used in each species induced similar changes in retinal fatty acids [46]. In the ERG studies, rats and guinea pigs fed with omega-3 deficient diets had retinal DHA levels reduced by 30–65% in comparison with omega-3 sufficient control animals [47]. In the rhesus monkeys, retinal DHA was reduced by 50% at birth and by 80% at 2 years of age [43]. In each of the animal studies, the fall in retinal DHA was compensated for by an increase in retinal 22:5n-6 and, to a lesser extent, 22:4n-6 (AA).

## 4. Protective Role of DHA in the Retina

There is an abundance of literature suggesting a protective role of DHA in the retina, while nutrition is associated with AMD [48–50]. Some of the mechanisms of protection could be relevant for the purpose of prevention of AMD. 

### 4.1. Anti-Inflammatory Role

It is possible that the anti-inflammatory effect of omega-3 also plays a positive role within the retina. The most significant change in the retinal phospholipids fatty acid composition after omega-3 intake is a large increase in the EPA/AA ratio [51]. In several tissues including the eye, the presence of increased levels of omega-3 among phospholipids has been reported to inhibit competitively the formation of cyclooxygenase and lipoxygenase products of AA and to promote the formation of the less potent omega-3 series eicosanoids, resulting in anti-inflammatory effects [52, 53]. An omega-3 diet also induces a decrease in the production of platelet activating factor (PAF) [54]. Moreover, DHA modulates the effects of C-reactive protein, homocysteine, intercellular adhesion molecules-1 (ICAM-1), and vascular adhesion molecules (VCAM-1) [55, 56]. Chen et al. examined the anti-inflammatory effects of DHA on adhesion molecule expression such as ICAM-1 and VCAM-1 induced by cytokines in human retinal vascular endothelial cells [55]. They suggest that a decrease in DHA leads to the increase in cytokine-induced proinflammatory signaling in retinal microvasculature.

### 4.2. Antiangiogenesis Role

Many sight-threatening diseases have two critical phases, vessel loss followed by hypoxia-driven destructive neovascularization. The influence of omega-3- and omega-6-polyunsaturated fatty acids on vascular loss, vascular regrowth after injury, and hypoxia-induced pathological neovascularization was recently studied by Connor et al. in a mouse model of oxygen-induced retinopathy [56]. They showed that increasing omega-3 PUFA tissue levels by dietary or genetic means decreased the avascular area of the retina by increasing vessel regrowth after injury, thereby reducing the hypoxic stimulus for neovascularization. The bioactive omega-3 PUFA-derived mediators (neuroprotectinD1, resolvinD1, and resolvinE1) protected against neovascularization. These findings indicate that increasing the sources of omega-3 PUFA or their bioactive products reduces pathological angiogenesis.

### 4.3. Antiapoptotic Role

In rat retinal cells cultured in a serum-free medium, the addition of DHA to the cultures prevents the selective apoptotic death of photoreceptors [57]. Compared with other fatty acids, DHA was not only the most effective in promoting photoreceptor survival, but also the only fatty acid to decrease the number of apoptotic nuclei. In cultures lacking DHA, photoreceptors develop normally for 4 days and then start an apoptotic pathway leading to extensive degeneration of these cells by day 11 [58]. In addition, DHA advances photoreceptor differentiation, promoting opsin expression and inducing apical differentiation in these neurons. Furthermore, mitochondrial damage associated with photoreceptor apoptosis is markedly reduced upon DHA supplementation, suggesting that a possible mechanism of DHA-mediated photoreceptor protection might be the preservation of mitochondrial activity. The authors hypothesized that a critical amount of DHA in mitochondrial phospholipids might be required for proper functioning of these organelles, which in turn might be essential to avoid cell death. It is notable that this protective effect of DHA can be synergic with the neuroprotective effect of lutein and zeaxanthin [59]. 

Rotstein et al. analyzed rat retinal cells in 3-day cultures treated with the oxidant paraquat, with or without DHA [57]. They demonstrated that DHA activates intracellular mechanisms that prevent photoreceptor loss, and modulates the levels of pro- and antiapoptotic proteins of the Bcl-2 family, protecting photoreceptors from oxidative stress. Similarly, it was demonstrated a diet rich in DHA plays a role in protection against MNU-induced photoreceptor cell apoptosis in the rat retina [60]. 

The synthesis of a specific mediator generated from DHA, the neuroprotectin D1, (NPD1), was recently demonstrated [61]. NPD1 has interesting properties: it counteracts tumor necrosis factor alpha oxidative-stress-triggered apoptotic RPE DNA damage, it upregulates antiapoptotic proteins expression. Moreover, NPD1 protects RPE cells from oxidative-stress-induced apoptosis, and plays anti-inflammatory and neuroprotectine roles [39, 62]. This lipid mediator therefore may indirectly contribute to photoreceptor cell survival.

### 4.4. Protection from Neurotoxicity

The protective effect of DHA against transient retinal ischemia was investigated by increasing intraocular pressure in rats. When DHA is administered before experimental ischemia, it diminished pressure-induced retinal damage [61]. The recovery of electroretinographic amplitudes after reperfusion is significantly greater in DHA-pretreated eyes than in non-pretreated eyes. Based on their experiments, Murayama et al. concluded that DHA prevents sensory retina from ischemic-reperfusion cell damage not only by inhibiting the formation of hydroxyl radicals, but also by reducing the apoptotic responses [61]. DHA is in turn involved in the regulation of expression of apoptosis mediators (Bcl-2 and Bax) preserving mitochondrial membrane potential and inhibiting caspase activation [63, 64].

Miyauchi et al. investigated the ability of DHA to protect the retina from kainic acid- (KA-) induced retinal damage [65]. Both morphologic and functional attenuations were significantly less in the DHA-supplemented rats than in control rats. In this experiment, DHA was able to partially prevent retinal degeneration induced by kainic acid. Hence, DHA promotes photoreceptor survival and differentiation by activating the same signaling pathways triggered by peptidic trophic factors.

Yee et al. investigated the role for omega-3 polyunsaturated fatty acid supplements in rats with diabetic retinopathy [66]. A partial improvement was evident in the oscillatory potentials at electroretinogram, most likely secondary to the larger photoreceptor output.

### 4.5. Enhancement of RPE Acid Lipase Omega-3

The RPE lysosomal acid lipase is an enzyme involved in hydrolysis and thus the clearance of intralysosomal RPE lipids. Omega-3 fish oil, delivered to the retinal pigment epithelium (RPE) by circulating low-density lipoproteins (LDLs), enhances considerably the effects of RPE lysosomal acid lipase [67]. This was shown *in vitro* by analysis of RPE cells from monkeys. From these experiments, Elner hypothesized that diets rich in fish oil-derived omega-3 fatty acids, by enhancing acid lipase, may reduce RPE lipofuscin accumulation, RPE oxidative damage, and then could slow down development of AMD [67].

### 4.6. Omega-3 and Peroxidation in the Retina

In the retina, lipid peroxidation is thought to be a major mechanism contributing to light-induced lesions. Lipid peroxides, found in the Bruch's membrane, have been shown to induce neovascularization by inducing expression of a cascade of angiogenic cytokines [68]. The increase in amount of peroxidized lipids with age, combined with their vasogenic potential, suggests that it may play a role in the etiology of neovascular AMD, particularly choroidal neovascularization. The question of peroxidizability of ROS with supplementation in DHA has been raised, and several studies analyzed the levels of hydroperoxides in rod outer segments membranes with contradictory results [35, 69]. Owing to its high degree of unsaturation, with 6 double bounds, DHA is vulnerable to peroxidation. This is compounded by exposure to light, high oxygen tension, and high concentrations of retinol [70]. Depletion of retinal DHA by ALA deficient feeding reduces the susceptibility of the rats ROS to acute light damage, which appears to be related to the relative levels of DHA [71, 72]. Light-induced stress to the retina in these studies was carried out by exposing rats to intermittent light with approximately 100 times luminous intensity over the control. In a dietary supplementation study on young rats fed with high levels of DHA, it was observed *in vitro* that ROS membranes submitted to UV intense irradiation may form phospholipid hydroperoxides more efficiently with higher DHA content than with low DHA [69]. However, *in vivo*, at normal light conditions, there was no increasing tendency in the hydroperoxide levels of ROS membranes containing high content of DHA, which implies that DHA supplementation does not much affect the peroxidizability of ROS membranes *in vivo* [69]. 

Other studies support the hypothesis of a partial protective effect of DHA despite increased peroxidation. Reme et al. investigated the effect of fish oil on acute light-induced photoreceptor damage in rats *in vivo* [51]. They observed an enhanced susceptibility to lipid peroxidation in the isolated retina of fish oil fed rats. However, fish oil fed rats showed less damage at the base of the rod outer segments, suggesting that omega-3 does not enhance the susceptibility to acute ROS disk disruptions in the rat retina but exerts a partial protective effect. Furthermore, Reinboth et al. demonstrated that light elicits the *in vitro* release of DHA from photoreceptor phospholipids in rats, and proposed that this release may serve a protective role in the retina by suppressing ARA-derived eicosanoids associated with inflammatory responses [73].

Sunada et al. [74] investigated how dietary DHA affects the generation of lipid peroxides in rat retina under oxidative stress in diabetes with/without vitamin E (VE) deficiency. They found, in rats with VE deficiency, dietary DHA increased serum and liver lipid peroxide levels but not in the retina. These results suggest that dietary DHA does not necessarily promote lipid peroxidation in the retina even under high oxidative stress. 

In parallel to DHA, AA also has a high potential of peroxidability, 3 times greater than that of ALA [75], mediated by cyclooxygenase and lipooxygenase products of AA [76]. The F2 isoprostanes (F2-IPs) constitute a novel class of prostaglandin-F2- (PGF2-) like compounds that are produced by the non-cyclooxygenase peroxidation of AA [77–79]. These mediators are reliable markers of oxidative stress both *in vitro* and *in vivo* studies [80, 81]. It was shown in retina of rats that dietary fish oil increases the concentration of DHA and decrease the concentration of AA in ROS [51]. It is thus possible that DHA supplementation could reduce the levels of F2-IPs in the retina. 

Similar to the issue of peroxidation, the percentage of trans isomers of omega-3 could influence visual function. Acar et al. performed ERG in rats feed with ALA in its natural form (cis-isomer) or partially isomerized (*cis* and trans isomers) [82]. Dietary trans ALA altered the fatty acid composition of retinal phospholipids by significantly increasing the Delta19 trans isomer of DHA. Moreover, dietary trans isomers of ALA significantly decreased the b-wave amplitude of the ERG by 9 months of feeding, suggesting that long-term intake of small amounts of trans isomers of ALA could disturb visual function. The authors hypothesized that it is at least as important to prevent the consumption of trans isomers of ALA as to correct the unbalanced omega-6/omega-3 ratio by increasing the level of the natural isomer [17]. 

## 5. AMD and Omega-3

### 5.1. Epidemiology of AMD and Omega-3 Consumption

Some populations such as the Japanese have reported an unusual low incidence of AMD among their elderly in the past [83]. However, in the last decades, physicians in Japan have reported a significant increase in incidence of AMD in their clinics, particularly for exudative AMD [22]. It is also notable that the form of AMD in Japan seemed subtly different from the ones described in Western countries, with less observed drusen. This population is genetically homogenous, poorly mixed, without modification of the genetic background. However, environmental factors changed including the food habits of the population, eating less fish, and adopting an occidental diet. The omega-6/omega-3 ratio in Japan is substantially lower than in the UK and USA. The Japanese figure in 1985 (3.9 : 1) was higher than it was in 1960 (2.9 : 1) because of a trend towards lower fish consumption [84]. Interestingly, a study analyzed the AA and DHA red blood cells levels in a small Japanese study population affected with AMD (*n* = 11) compared with controls (*n* = 10). No significant variations were observed between the two groups in terms of plasma levels of AA, DHA, palmitic, palmitoleic, oleic, and linoleic acids. However, the number of patients was small and there was no clear classification of AMD [72]. 

Similarly, in Iceland, the phenotype of ARM and AMD is different from most other North European communities [85]. This difference is expressed by the higher prevalence of geographic atrophy (more than 80% of advanced cases) in Iceland compared with other populations. The predominance of atrophic form of AMD could be genetically explained by homozygosis (common ancestor). On the other hand, the relative low prevalence of exudative forms could be explained by environmental factors such as nutrition. The Icelandic population is relatively well nourished. According to the United Nations Food and Agriculture Organization, no European nation eats more fish per capita than Icelanders although their consumption of green vegetables and fruits is relatively low by European standards [86]. Unfortunately, the possibility that Icelandic emigrants might develop more wet AMD has not yet been investigated, which would assess the roles of nutrition and other environmental factors versus genetic protection. 

### 5.2. Fish Consumption Prevalence and Incidence of AMD

The hypothesis of a relationship between omega-3 and AMD has been evaluated in many studies of dietary intake [87, 88]. Sanders et al. performed a matched-control study of PUFAs content of plasma and RBCM on 65 AMD patients and 65 controls [89]. They did not observe any difference in concentrations of DHA between AMD patients and controls in the serum or in the RBCM. The sample size was small and most of the AMD patients included in this study were affected with drusen and/or pigment changes, but only two were affected with neovascular AMD. 

Studies which investigated the relationship between consumption of omega-3, based on frequency food questionnaire (FFQ), and the occurrence of AMD. It is notable that these different studies, analyzing different populations, all suggest a protective effect of omega-3 fish oil. 

The first report of a statistical analysis showing the effect of the relationship between dietary omega-3 fatty acids and AMD was presented at the ARVO meeting in 1994 [90]. The Ancillary Dietary Study of the Eye Disease Case-Control Study included 349 patients with exudative AMD and 504 unaffected controls, and demonstrated a strong positive association between exudative AMD and consumption of all fats and an inverse association with fish oil [86]. A strong association was observed between higher linoleic acid intake and the disease, with an odd ratio (OR) in multivariate analyses for the 5th versus the 1st quintile of 2.0 (*P* = 0.02). Conversely, intake of omega-3 fatty acids showed an inverse relationship with AMD in demographically adjusted analyses (OR: 0.59, *P* = 0.01), but the significance of the association decreased after multivariate analyses, with an odd ratio of 0.75. In individuals consuming low omega-6 (linoleic acid) and high omega-3, the relative risk of exudative AMD was 0.60. 

In 1995, Mares-Perlman et al. [87] reported the results of the Beaver Dam Eye Study, that included 1968 participants, including 314 early ARM individuals but only 30 late AMD patients (21 wet AMD and 9 geographic atrophy). Intake of seafood, considered as a marker of intake of omega-3 fatty acids, was unrelated to early or late macular degeneration. The calculations were made in g/4200 kJ, comparing quintile 5 to quintile 1. The odds ratio for early ARM was 0.9 (*P* = 0.3) and odds ratio for late ARM was 0.8 (*P* = 0.4). However, the low number of patients affected with late AMD limited the power of this study. 

In the Blue Mountains Eye Study population, a clear association was observed between higher frequency of intake of fish (>1 per week) with decreased risk of late age ARM [91], compared with lower frequency of consumption (<1 per month) with an odds ratio approximately at 0.5 [92]. More precisely, when they compared the higher frequency (≥5 per week) to the lower frequency of intake of fish (<1 per month), the odds ratio adjusted for age, sex, smoking status, and familial history of late ARM was at 0.46 for late ARM. To investigate the longitudinal associations between dietary fat and incident age-related ARM, 3654 persons who participated in the Blue Mountains Eye Study were reexamined after 5 years [93]. Participants with the highest versus lowest quintiles of omega-3 polyunsaturated fat intake had lower risk of incident early ARM (OR: 0.41), whereas fish consumption at least 3 times per week could reduce the incidence of late ARM (OR: 0.25).

The third National Health and Nutrition Examination Survey (NHANES III) described similar associations between fats and AMD in a cross-sectional study [94]. The AMD status was based on fundus photography of one eye in 7883 participants, and the intake of fats was based on a FFQ. Among them, 644 were classified as early ARM and a small number of individuals with advanced AMD was observed (*n* = 53). After adjustment for age, race, eye color, and sedentary lifestyle, the odd ratio for early ARM was 1.4 (*P* = 0.10) comparing the 5th versus the 1st quintile. They did not observe a similar relationship for advanced AMD but the small number of these patients limited the power of this study. Nevertheless, they found that consumption of fish more than once a week compared with once a month or less was associated with odd ratio of 1.0 for early ARM and 0.4 for late ARM (*P* = 0.10) after adjusting for age and race. 

In the Nurses' Health study [95], total fat intake was also associated with an increased risk of AMD. Linolenic acid was positively associated with risk of AMD; the highest quintile of intake was associated with a 49% increased risk compared with the lowest quintile. Adjustment for total fat and trans-unsaturated fat did not attenuate the RR for AMD. In contrast, DHA intake had an inverse relation with AMD; the pooled multivariate relative risk for the highest quintile of DHA intake compared with the lowest was 0.70. Participants who ate fish >4 times per week had a lower risk of AMD than those who consumed it <4 times per month (RR: 0.65). More specifically, the pooled relative risk of participants who ate canned tuna more than once per week compared with those who consumed it less than once per month was 0.61.

The US Twin Study of Age-Related Macular Degeneration [50] also investigated risk for AMD, according to dietary fat intake in 221 twins with AMD (intermediate or late stages) and 459 twins with no maculopathy or early signs. The authors found that dietary omega-3 fatty intake was inversely associated with AMD, comparing the highest versus. lowest quartile (OR: 0.55). Reduction in risk of AMD with higher intake of omega-3 fatty acids was seen primarily among subjects with low levels of omega-6 fatty acids. 

The EUREYE study [96], analysing the diet of European elderly, found that eating oily fish at least once a week compared with less than once a week is associated with a halving of the odds of neovascular AMD (OR: 0.47; *P* = 0.003). Compared to the lowest quartile there was a significant trend for decreased odds with increasing quartiles of either DHA or EPA (odds ratios in the highest quartiles were OR: 0.32, *P* = 0.04 for DHA, and OR: 0.29, *P* = 0.02 for EPA). 

The POLANUT study also recently assessed the associations of dietary fat with the risk of ARM, in 832 subjects aged 70 years or more of the POLA (Pathologies Oculaires Liées à l'Age) cohort study [97]. Subjects in the highest quintile of energy-adjusted total, saturated, and MUFA intake were associated with increased risk for ARM (OR: 4.74, *P* = 0.007; OR: 2.70, *P* = 0.04; OR: 3.50, *P* = 0.03, resp.), by comparison with subjects in the lowest quintile. Total PUFA was not significantly associated with ARM (OR: 1.02, 95% CI: 0.29–3.53, *P* = 0.94), but fatty fish intake (more than once a month versus less than once a month) was associated with a 60% significant reduction in risk for ARM (OR: 0.42; *P* = 0.01). This study is consistent with increased risk of ARM in subjects with high dietary intake of fat (in particular high MUFA intake) and conversely suggests a potential protective effect of long-chain 3 PUFA.

Hodge et al. [98] reviewed the literature without restriction and concluded that the efficacy of dietary and/or supplemental omega-3 fatty acids in preventing AMD was neither clearly supported nor refuted by the world literature. However, despite the differences in methodology and populations, all of these studies suggested a protective role of DHA for AMD, particularly for exudative AMD. Moreover, the inverse relationship between omega-3 fish oils highly contrasts with the positive association between other types of fat and AMD [99]. Further studies with prospective cohort designs and randomized clinical trials are needed.

The AREDS group also investigated the role of omega-3 PUFAs in the occurrence of exudative AMD [100]. They observed a significant lower relative risk of neovascular AMD associated with higher consumption of omega-3 (the 5th versus the 1st quintile), with an odd ratio at 0.61 (*P* < 0.01). This inverse relationship was even more significant when analyzing specifically the higher versus. lower consumption of DHA, with an odds ratio at 0.54 (*P* < 0.004). Higher fish consumption, both total and broiled/baked, was also inversely associated with NV AMD (OR: 0.61 and OR: 0.65, resp.). Conversely, dietary arachidonic acid was directly associated with NV AMD prevalence (OR: 1.54). 

### 5.3. Fish Consumption and Progression of AMD

A prospective cohort study based on an FFQ evaluated the potential role of fat intake on the progression from early or intermediate forms of AMD to advanced forms of AMD. This study suggested a protective effect of fish intake among individuals with lower linoleic acid intake [49]. There was a positive association between higher saturated, monounsaturated, polyunsaturated fat, and transunsaturated fat intake and progression of AMD, with multivariate RR's, respectively, of 2.09 (*P* = 0.08), 2.21 (*P* = 0.04), 2.28 (*P* = 0.04), 2.39 (*P* = 0.008). The analysis of relatives risks for progression to advanced AMD by frequency of fish intake did not reveal a significant difference between individuals eating less than one serving of fish per week and individuals eating more than once a week (RR: 0.88; *P* = 0.42). However, when subjects were stratified by linoleic acid, the major source of omega-6 fatty acids, fish intake decreased the risk of AMD progression when LA was below the median, with a multivariate RR of 0.36 (*P* = 0.045). Consumption of nuts also seemed to have a protective effect on the progression of AMD, with a multivariate RR of 0.60 (*P* = 0.05) when comparing individuals eating nuts more than once a week versus. none. This could be explained by the presence of linolenic acid (ALA), precursor of DHA, in nuts. The main advantage of this study is the large size of patients and its prospective design. 

In contrast to all of the other studies to date, an Australian prospective study found a positive association between intake of omega-3 and progression of AMD (OR: 2.56, *P* = 0.03) [101]. This study included 254 individuals, followed during 7 years. In their discussion, the authors suggest the possibility that participants of this study adopted a more healthy diet, having been aware of their AMD status at the beginning of the study.

### 5.4. Interventional Studies

To the best of our knowledge, very few clinical trials investigated the role of oral supplementation with omega-3 for the prevention of AMD. A French study called NAT-1 (Nutritional AMD Treatment, phase 1) evaluated the feasibility of a prospective interventional study for oral supplementation with fish oil in a homogenous AMD population [102]. A homogeneous group of 38 patients affected with drusenoid pigment epithelial detachment in one eye (PED) without choroidal new vessels (CNV) was studied. The population was divided into two subgroups, with (*n* = 22) or without (*n* = 16) omega-3 supplementation (EPA: 720 mg/day and DHA: 480 mg/day), over a 6-month period. A significant blood enrichment in EPA in serum (S) and in red blood cell membranes (RBCMs) (EPA-S: 2.20% versus 0.79%, *P* < 0.0001 and EPA-RBCM: 2.24% versus 0.85%, *P* < 0.0001) and in DHA (DHA-S: 2.47% versus 1.56%, *P* < 0.0001 and DHA-RBCM: 6.47% versus 4.67%, *P* < 0.0001) was observed in the treated group at the 6th-month visit. The absorption of omega-3 PUFAs did not seem to be altered in this aged population. During this short period of time, no progression was observed in the size of the drusenoid PED, in the areas of atrophy and no evolution to CNV was noted in any of the two groups. Neither side effects nor dropouts were observed. NAT-1 supported for the feasibility of a long-term double-masked prospective study in an AMD population in order to evaluate the potential benefit from oral supplementation with DHA. 

An Italian study analyzed the benefit of supplementation with Mitotrop, a product containing acetyl-L-carnitine, omega-3 and coenzyme Q10 [103, 104]. A total of 106 patients with early AMD were included within a period of 12 months of treatment. Visual function was evaluated by best corrected visual acuity and by analysis of 10 degree visual field mean defect. In the treated group, the visual function showed slight improvement and the divergence between treated and control groups became more marked with time, but not statistically significant. The authors supported that the association of these compounds may improve retinal function in early AMD. 

A study conducted at 5 independent study sites, the TOZAL study, investigated the change from baseline in visual function at 6 months in 37 subjects with atrophic AMD treated with a targeted nutritional supplement (taurine, omega-3 fatty acids, zinc, antioxidant, lutein) [105]. There was a statistically significant improvement in visual acuity from baseline compared to a placebo-cohort constructed from the literature that was matched for inclusion and exclusion criteria (*P* = 0.045). The results of the TOZAL study agree with the LAST [95] and CARMIS studies and are predictive for positive visual acuity outcomes in clinical trials of omega-3 supplementation.

The OPAL study [106] has been recently launched to asses if an increased dietary intake of n-3 PUFAs will have a positive effect on cognitive performance in older people in the UK. To test this hypothesis, a double-blind randomised placebo-controlled trial will be carried out among adults aged 70–79 years in which the intervention arm will receive daily capsules containing n-3 PUFAs (0.5 g/day DHA and 0.2 g/day EPA), while the placebo arm will receive daily capsules containing olive oil. The main outcome variable assessed at 24 months will be cognitive performance and a second major outcome variable will be retinal function. 

The NAT-2 is a monocentric prospective, interventional, randomized, and double-masked trial conducted in France. Criteria for inclusion are presence of CNVs in one eye only, and presence of drusen in the study eye (*n* = 298 AMD patients). A complete ophthalmologic examination is performed including best-corrected visual acuity, multifocal ERG, fluorescein angiography (FA), OCT, as well as a complete lipid profile including cholesterol, triglycerides, LC-PUFAs in the serum and red blood cell membranes (RBCM), and ApoE genotype. All individuals, in the treated and untreated groups are followed for 3 years. The primary outcome is the occurrence of choroidal neovascularization in the study eye. The secondary parameters are the evolution of the visual function (visual acuity and multifocal ERG), the occurrence of atrophy, and the evolution of drusen (quantity and area). Preliminary results of NAT–2 revealed that HDL level mean (95% CI) was particularly elevated in the NAT-2 population (298 patients from the NAT-2 study at baseline) (1.74 mmol/L) versus healthy age and sex-matched French population (2030 individuals without any drusen or macular lesion) (1.37 mmol/L) (*P* < 0.0001) [107]. This finding is in agreement with a recent study [74] reporting that increasing HDL cholesterol is inversely related to incident late AMD (RR per standard deviation [SD] increase, 0.74). Mean MUFAs level was also elevated in the NAT-2 population (24.9%) versus non-AMD population (20.4%) (*P* < 0.0001). Mean omega-6, omega-3, and DHA levels were lower in the NAT-2 population (resp., 32.4%, 2.34%, and 1.28%) versus non-AMD population (resp., 37.2%, 3.06%, and 1.89%) (*P* < 0.0001). Therefore, high HDL levels and low PUFAs levels were observed particularly low DHA levels, in exudative AMD patients. These findings support the potential benefit of DHA supplementation in AMD patients. 

The AREDS-2 study is a multicenter-randomized trial designed to assess the effects of oral supplementation of high doses of macular xanthophylls (lutein and zeaxanthin) and/or omega-3 LCPUFAs (DHA and EPA) on progression to advanced AMD [108]. This objective will be accomplished by collecting and assessing the data on approximately 4000 AREDS 2 participants aged from 50 to 85 years, who at the time of enrolment have either (1) bilateral large drusen or (2) large drusen in one eye and advanced AMD (neovascular AMD or central geographic atrophy) in the fellow eye. All participants will be offered additional treatment with the study formulation used in AREDS. For those who elect to take this additional supplement, which is now considered the standard of care, further randomization may occur to evaluate the possibility of deleting beta-carotene [109] and decreasing the original levels of zinc in the formulation for the treatment of AMD, if consent is obtained. Study participants are therefore assigned randomly to take one of the following study supplements on a daily basis: (1) placebo, (2) Lutein/zeaxanthin (10 mg/2 mg), (3) DHA/EPA (1 g), or (4) Lutein/zeaxanthin and DHA/EPA (10 mg/2 mg and 1 mg), with a secondary randomization involving modified doses of AREDS 1 supplements. 

## Figures and Tables

**Figure 1:**